# Model Context Protocol: The unexpected catalyst of a bioinformatics interoperability revolution

**DOI:** 10.1371/journal.pcbi.1014543

**Published:** 2026-08-31

**Authors:** Nathan C. Sheffield

**Affiliations:** 1 Department of Genome Sciences, School of Medicine, University of Virginia, Charlottesville, Virginia, United States of America; 2 Department of Biochemistry and Molecular Genetics, School of Medicine, University of Virginia, Charlottesville, Virginia, United States of America; 3 Department of Biomedical Engineering, School of Medicine, University of Virginia, Charlottesville, Virginia, United States of America; 4 School of Data Science, University of Virginia, Charlottesville, Virginia, United States of America; Yale School of Public Health, UNITED STATES OF AMERICA

## Introduction

### Fragmentation in bioinformatics

From humble beginnings of the first protein databases with just dozens of protein sequences [1,2], the field of bioinformatics exploded alongside advances in sequencing technology. Yet this rapid growth came with a critical challenge: every research group invented its own databases, terms, and formats from scratch, creating a fragmented ecosystem with tricky integration challenges [3]. The examples are legendary among bioinformaticians: lack of coherence among bioinformatic databases and data formats is almost a meme. For example, the ubiquitous FASTA format lacks formal specification, leading to incompatible interpretations by different parsers. FASTQ, the standard for sequencing data, exists in several incompatible variants with different quality score encodings [4,5]. The General Feature Format (GFF) has spawned many versions, with a 2010 study noting there are “as many flavours as tools producing it” [6]. Formats like VCF (Variant Call Format) struggle with incompatible extensions added by different variant callers [7].

These challenges are not limited to file formats. Gene and protein identifiers vary between databases and species [3]. Transcript annotation models differ not only between reference providers but also across genome releases, leading to mismatches in coordinate systems and exon-intron structures that can alter downstream analyses [8]. Reference genomes use different naming schemes for chromosomes [9,10]. Ontologies and controlled vocabularies such as the Gene Ontology have helped, but adoption is uneven, and semantic mismatches still create integration headaches.

The proliferation of incompatible representations wasn’t mere technical incompetence. Academic incentives have always favored creating novel tools over improving existing ones. Unfortunately, grant funding rewards innovation, not standardization. Furthermore, geographic isolation and independent biological focus mean research groups work in silos, each optimizing formats for its specific computational constraints. And fundamentally, the biology behind different species, and even within a species, is complicated and varied, adding layers of complexity to any attempt at universal standards. Given the relative newness of bioinformatics, without a history of solid concepts to build on, the field has moved quickly and as a whole, didn’t pause much in the early days to establish standards.

### The persistent interoperability crisis despite recent progress

The past decade has witnessed significant efforts to address this fragmentation. The Global Alliance for Genomics and Health (GA4GH) has developed dozens of approved standards spanning data formats, APIs, and policy frameworks [9,11–13]. The FAIR (Findable, Accessible, Interoperable, Reusable) data principles have gained widespread acknowledgment [14]. Workflow standardization efforts through Common Workflow Language (CWL), Workflow Description Language (WDL), and Nextflow have emerged [15]. A variety of efforts are creating new API-oriented data services [16]. But despite these initiatives, the reality remains challenging [17–22]. Clinical genomics faces particular challenges, with electronic health record integration remaining a major bottleneck, and much clinical genomic data going unused due to siloed systems [23,24]. Federated platforms face significant interoperability hurdles [25], and the semantic interoperability that would ensure consistent meaning across shared data remains unsolved [26].

The voluntary nature of standards adoption creates a chicken-and-egg problem. Institutions hesitate to invest in standardization without clear benefits and existing users, yet benefits only materialize with widespread adoption. These significant cultural and technical barriers continue to hinder standardization [27].

### Secondary analysis benefits of interoperability

But the need for interoperability is only increasing. Genomic datasets are growing exponentially: the Sequence Read Archive expanded from 3.6 petabases in 2015 to over 50 petabases by 2024 [28]. As datasets grow, so too grows the cost of either converting formats or maintaining multiple versions to satisfy different analysis needs. Many public data resources require controlled access, which requires interoperable query interfaces and ability to index files in a uniform way. The most important analyses now integrate multiple data types and multiple data sources, which are frequently distributed across institutions and countries; analyzing them together requires shared protocols, identifiers, and formats. Without interoperability, these large, diverse resources remain fragmented and underused.

When interoperability works, the benefits are transformative [23,29–33]. But given the checkered history of biological data standardization and the continuing torrent of unstandardized data, how do we realize those benefits?

### The unexpected catalyst: Model Context Protocol

In the past 2 years, deep learning models have begun revolutionizing how we process biological data. Meta’s ESMFold predicts protein structures from sequences [34]; Google’s AlphaFold2 has transformed protein structure prediction [35]; Geneformer processes single-cell transcriptomes for context-aware analysis [36]. Like their NLP-oriented cousins the Large Language Models (LLMs), these biology-oriented models consume large training datasets in standardized formats. The need to train these models has provided a new incentive to curate datasets in standardized, interoperable formats [37]. This is a welcome step toward increased interoperability, and an important advance toward getting the most from biological data. However, I argue that the biological interoperability revolution of the coming decade will only partly be motivated by training biological models; instead, the most promising driver will come from another direction: the drive to apply LLMs to analysis of biological data. This direction is rapidly moving forward, most recently through a promising development in the AI community: The Model Context Protocol (MCP) standard.

Launched by Anthropic in November 2024, MCP services provide a universal interface for AI systems to access external resources [38]. In simple terms, MCP is a standard way for AI to plug into tools and databases, much like USB allows devices to connect to a computer without custom wiring. An MCP server is an application that runs either locally or on the web and provides, for example, “MCP tools” that can be invoked by an LLM. The MCP server may provide all kinds of capabilities through tools, such as looking up information in a local database, performing a web search, querying a remote API, or even taking action like triggering a job, running an analysis, calling a function, or sending a message.

**Fig 1:**
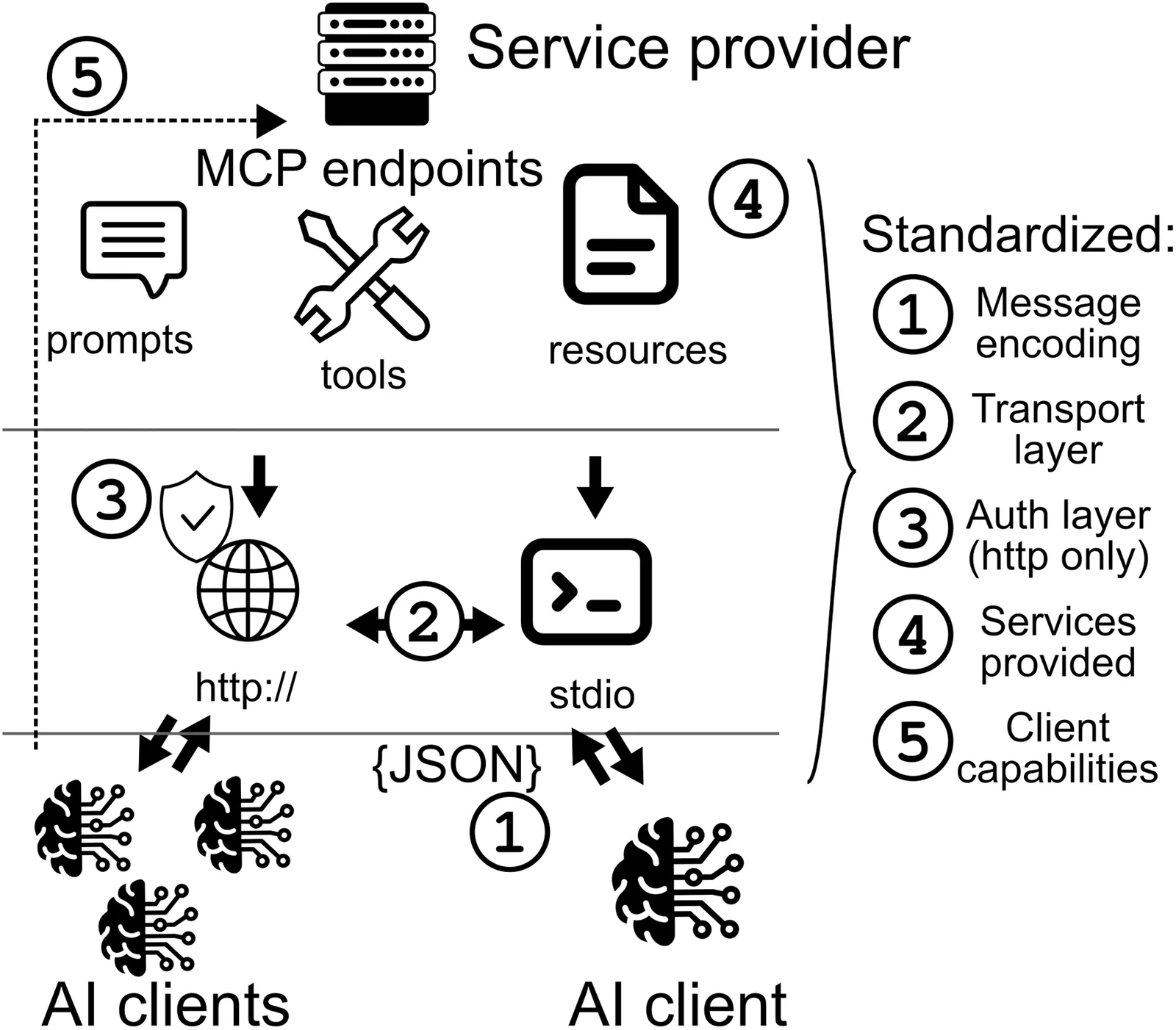
Model Context Protocol. The Model Context Protocol defines the interface between a service provider and AI client at 5 levels.

At its core, MCP is very simple. As of the 2025-11-25 specification, it defines the interface between the AI client and the MCP server at several levels (Fig 1): 1) a message encoding data format using JSON-RPC 2.0 for requests and responses; 2) a transport protocol using either standard in/out or streamable HTTP; 3) an optional OAuth 2.1-based authorization layer specifically for remote HTTP services; 4) definitions of entities that MCP servers expose (tools, prompts, and resources); and 5) capabilities that clients may optionally provide back to servers, such as “elicitation”, which allows a server to request structured information from a client, or “sampling,” which allows servers to request LLM inference from the client. The protocol is evolving rapidly; a major revision in 2026 makes the protocol core stateless and moves several capabilities into independently versioned extensions, and also deprecated “sampling” shortly after its introduction. The power of MCP is in standardization and discovery: Once a resource implements MCP, any AI system can access it through a standardized interface, eliminating the need for custom integrations. The capabilities afforded an LLM via MCP enables AI systems to act on information in real time, making MCP a key driver of the emerging agentic AI paradigm. Its support for dynamic capability discovery lets AI agents work with new data sources without reprogramming or retraining, a critical advantage in the fast-changing world of biological databases.

Already, biology-oriented MCP servers are proliferating: implementations like BioMCP, BioContextAI [39], MCPmed [40], galaxy-mcp, and the Holy Bio MCP Framework are integrating major databases including PubMed, UniProt, and ClinicalTrials.gov, making these biological data available to LLMs as external information. This rapid adoption demonstrates how MCPs are accelerating standardization of biological data for use by LLMs. The desire to use AI for biological reasoning creates bottom-up pressure for interoperability that top-down mandates never achieved.

Crucially, LLM-driven standardization benefits extend beyond AI applications. The same interoperable formats that will allow AI agents to access data via MCP will also facilitate traditional bioinformatics workflows and enable service-oriented biological data analysis that has been a difficult-to-reach goal for many years [3,16,25].

### The path forward: Embracing the AI-driven revolution

While much of the progress of LLMs in the past few years has been driven by improvements in the models themselves, that improvement trajectory will eventually asymptote. At some point, advances will be driven primarily through making more powerful tools and more granular data available to LLMs. This, in turn, will surface a new manifestation of the same old data interoperability challenge, but with a fundamental difference: the potential of LLM analysis on interoperable biological data tips the scales significantly more to the side of the benefits of interoperability. The bioinformatics community stands at a pivotal moment. Decades of well-intentioned but fragmented standardization efforts have achieved some success, but not fulfilled the complete vision of interoperable biological analysis. Now, the promise of AI-driven biological data analysis offers what deliberate planning could not: a powerful reason for universal adoption of API-oriented interoperable standards.

The revolution won’t happen overnight; legacy systems persist, novel data formats arise, and institutional inertia resists change. Yet the trajectory is clear: as more researchers experience the power of LLMs for biological analysis, demand for MCPs to augment AI systems will grow. Tools and databases that embrace these standards will thrive; those that don’t risk obsolescence.

So far, the biology-oriented MCPs are mostly restricted to high-level information, but the drive will continue in the direction of more and more granular data types as both LLM and MCP capabilities evolve. We will probably never reach a point where an LLM is loading millions of individual sequencing reads into context for analysis; but still, it could be valuable for an LLM to view samples of read data, investigate specific individual reads, or identify chunks of remote data for passing to other analysis tools. All of these applications are facilitated by MCP. As demand for LLM-accessible biological data increases, this will drive MCPs to evolve and expand, further incentivizing making biological data more machine-operable.

The field is moving quickly. MCP has become established as a critical piece of the puzzle of how to get interoperable data into an LLM context, but it is not the only piece. Other advances such as subagents, skills, standardized CLIs, and improved APIs are also significantly expanding the toolbox of the LLM. The point is: standards and practices that improve LLM-based data analysis of biological data will be transformational. MCP is an important step forward in this rapidly evolving landscape.

So, what can we do right now? We shift our daily work toward interoperability. The future of biological research belongs to those who provide and consume data via API. This means adopting standardized formats, implementing FAIR principles, and designing systems with interoperability as a core feature rather than an afterthought [16]. For data providers, it’s not so much the objective, but the incentives that have changed: make your data interoperable, not only for human or general programmatic consumption, but specifically for the benefit of current and future AI analysis. Deploy MCP services, starting with big-picture context for LLMs, but moving toward providing access to standardized schemas, summarized data, and references to data in chunks. For tool developers: build skill documentation for LLM-friendly tool use. For data analysts: use MCPs and provide feedback for data providers. Where LLMs struggle, identify their weaknesses and use this experience to improve MCPs, CLIs, and APIs to provide those capabilities.

The interoperability challenge that has constrained bioinformatics for decades is finally crumbling, not through committees or mandates, but through the promise of artificial intelligence. LLMs and MCPs are the catalysts for the interoperability revolution the field has long needed. The question isn’t whether this transformation will happen, but how quickly we can embrace it to unlock the full potential of biological discovery in the AI age. The reward for those who will embrace this potential will be the realization of bioinformatics’ founding vision: seamlessly integrated biological knowledge accessible to all researchers.
